# SP1-induced lncRNA-ZFAS1 contributes to colorectal cancer progression via the miR-150-5p/VEGFA axis

**DOI:** 10.1038/s41419-018-0962-6

**Published:** 2018-09-24

**Authors:** Xiaoxiang Chen, Kaixuan Zeng, Mu Xu, Xiuxiu Hu, Xiangxiang Liu, Tao Xu, Bangshun He, Yuqin Pan, Huiling Sun, Shukui Wang

**Affiliations:** 1 General Clinical Research Center, Nanjing First Hospital, Nanjing Medical University, Nanjing, 210006 Jiangsu China; 2 Medical College, Southeast University, Nanjing, 210009 Jiangsu China

## Abstract

Increasing long non-coding RNAs (lncRNAs) have been reported to play key roles in the development and progression of various malignancies. ZNFX1 antisense RNA1 (ZFAS1) has been reported to be aberrant expression and suggested as a tumor suppressor or oncogene in many cancers. However, the biological role and underlying molecular mechanism of ZFAS1, especially the miRNA sponge role of which in CRC remain largely unknown. We found that ZFAS1 expression was higher in CRC tissues, where it was associated with poor overall survival (OS), we also showed that ZFAS1 upregulation was induced by nuclear transcription factor SP1. Moreover, ZFAS1 and VEGFA are both targets of miR-150-5p, while ZFAS1 binds to miR-150-5p in an AGO2-dependent manner. Additionally, ZFAS1 upregulation markedly promoted as well as ZFAS1 knockdown significantly suppressed CRC cell proliferation, migration, invasion and angiogenesis, and the inhibitory effect caused by ZFAS1 knockdown could be reversed by antagomiR-150-5p. Lastly, we demonstrated that ZFAS1 knockdown inhibited EMT process and inactivated VEGFA/VEGFR2 and downstream Akt/mTOR signaling pathway in CRC. Our data demonstrated that SP1-induced ZFAS1 contributed to CRC progression by upregulating VEGFA via competitively binding to miR-150-5p, which acts as a tumor suppressor by targeting VEGFA in CRC.

## Background

Colorectal cancer (CRC) is one of the most common malignancies with high morbidity and morality, and more than one million new CRC cases were reported each year worldwide^1,2^. Recently, despite advanced treatments were used for CRC, including surgical resection, chemotherapy and radiotherapy, the overall survival (OS) rate of CRC patients have not increased substantially^3^. Notably, metastasis and postoperative recurrence are regarded as main causes of morality in these patients^4,5^. Therefore, to search for a reliable molecule involving in the progression and development of CRC is becoming urgent.

Long non-coding RNAs (lncRNAs), more than 200 nucleotides, are a class of no protein-coding RNAs and don’t contain open reading frame^6^. Recently, increasing lncRNAs have been reported to play critical roles in several biological process, such as cell senescence^7^, autophagy^8^, and cancer cell proliferation and metastasis^9^, and chemotherapy resistance^10^. Moreover, emerging aberrant expressions of lncRNAs have been implicated in various types of cancers, including colorectal cancer^11–13^. Additionally, lncRNAs could also serve as a endogenous RNA and sponge miRNAs which could regulate the expression of their target genes at post-transcriptional level, for example, lncRNA-MIR31HG exerts a competing endogenous RNA by sponging miR-193b and promotes tumor progression in pancreatic ductal adenocarcinoma^14^.

ZNFX1 antisense RNA1 (ZFAS1), located on chromosome 20q13, has been firstly reported to be dysregulated and suggested as a tumor suppressor gene in breast cancer^15^. Recent studies have shown that ZFAS1 was overexpression and identified to be involved in tumor growth and metastasis in several cancers, such as hepatocellular carcinoma (HCC)^16^, gastric cancer^17^, and osteosarcoma^18^. ZFAS1 has also been reported to be upregulated in CRC and downregulated of which suppresses proliferation, colony formation, and cell cycle of CRC cells^19^. However, the biological role and underlying molecular mechanism of ZFAS1, especially the miRNA sponge role of which in CRC remain largely unknown.

In our study, ZFAS1 was identified to be upregulated and associated with OS in CRC. We also revealed that ZFAS1 upregulation was induced by SP1. Moreover, downregulation of ZFAS1 could inhibit proliferation, metastasis and angiogenesis both in in vitro and in vivo. Moreover, our results also showed that miR-150-5p was identified as a negative regulator of vascular endothelial growth factor A (VEGFA) by directly targeting 3′UTR of VEGFA mRNA, resulting in decreased cell proliferation, migration and invasion and angiogenesis. Furthermore, our results also indicated that ZFAS1 functioned as an endogenous sponge to increase the expression of VEGFA through directly binding to miR-150-5p, which is involved in the activation of EMT and Akt/mTOR signaling way. Our findings uncover the role of ZFAS1 as a regulator of CRC progression, and sheds new light on our understanding of lncRNAs-mediated malignancy progression.

## Methods

### Cell culture and treatment

Human CRC cell lines HCT116, HCT8, HT29, SW620, SW480 and DLD-1, and corresponding normal colonic epithelial cell (FHC) were purchased from ATCC. These cells were cultured in Dulbecco’s Modified Eagle Medium (DMEM, Gibco, USA) with 100 U/ml penicillin, 0.1 mg/ml streptomycin, and 10% fetal bovine serum (FBS, Gibco,USA) at 37 ℃ supplied with 5% CO_2_ atmosphere.

### Patient samples

A total of 112 fresh CRC tissues and their corresponding adjacent normal tissues (ANTs) were obtained from patients with CRC who received radical surgery in Nanjing First Hospital, Nanjing Medical University between January 2001 and December 2007. None of the patients achieve system treatment before surgery. All these samples were immediately snap frozen in liquid nitrogen and stored at −80 ℃ until RNA extraction. Informed consent were obtained from all these patients and this study was approved by the Institutional Review Board of Nanjing First Hospital, Nanjing Medical University. Clinicopathological characteristic of these patients are listed in Table 1.

**Table 1:** Correlation between ZFAS1 expression and different clinical characteristics

Characteristics	*n* = 112	ZFAS1 expression		*P* value
		Low (%) (*n* = 56)	High (%) (*n* = 56)	
Gender				0.691
Male	74 (66.1%)	36 (64.3%)	38 (68.9%)	
Female	38 (33.9%)	20 (35.7%)	18 (31.1%)	
Age(years)				0.687
<60	36 (32.1%)	17 (30.4%)	19 (33.9%)	
≥60	76 (67.9%)	39 (69.6%)	37 (66.1%)	
Tumor location				0.346
Colon	59 (52.3%)	32 (57.1%)	27 (48.2%)	
Rectal	53 (47.7%)	24 (42.9%)	29 (51.8%)	
TNM stage				<0.001
I–II	65 (58.0%)	42 (75.0%)	23 (41.1%)	
III–IV	47 (42.0%)	14 (25.0%)	33 (58.9%)	
Differentiation				0.242
Low	27 (24.1%)	13 (23.2%)	14 (25.0%)	
Moderate	70 (62.5%)	32 (57.1%)	38 (67.9%)	
High	15 (13.4%)	11 (19.7%)	4 (7.1%)	
PT stage				0.008
T1–T2	13 (11.6%)	11 (19.6%)	2 (3.8%)	
T3–T4	99 (88.4%)	45 (80.4%)	54 (96.4%)	
PN stage				0.691
N0	66 (58.9%)	32 (57.1%)	34 (60.8%)	
N1	31 (27.7%)	16 (28.6%)	15 (26.7%)	
N2	15 (13.4%)	8 (14.3%)	7 (12.5%)	
Distant metastasis				0.003
M0	96 (85.7%)	53 (94.6%)	43 (76.8%)	
M1	16 (14.3%)	3 (5.4%)	13 (23.2%)	
Tumor size				0.702
<5 cm	66 (58.9%)	32 (57.1%)	34 (60.7%)	
≥5 cm	46 (41.1%)	24 (42.9%)	22 (39.3%)	
CEA				0.239
<10 ng/ml	72 (64.3%)	33 (58.9%)	39 (69.6%)	
≥10 ng/ml	40 (35.7%)	23 (41.1%)	17 (30.4%)	
CA199				0.345
<10 U/ml	51 (45.5%)	23 (41.1%)	28 (50.0%)	
≥10 U/ml	61 (54.5%)	33 (58.9%)	28 (50.0%)	

### Stable cell line construction and cell transfection

ZFAS1 siRNA lentivirus (siZFAS1-1, siZFAS1-2, siZFAS1-3, siZFAS-4) were obtained from Applied Biologic Materials, Inc (Canada) to knock down the expression of ZFAS1 and a scramble siRNA GFP Lentivector (scramble) was performed as a control, the siRNA sequence was listed in Supplementary materials and methods. Two CRC cell lines HCT116 and HCT8 were infected and stable cells were selected by treatment of puromycin (10 µg/ml). Small interferece RNA (siSP1-1, siSP1-2) targeting SP1 were chemically synthesized (Applied Biologic Materials, Inc, Canada), and the squence was listed in Supplementary materials and methods.

AgomiR-150-5p (miR-150-5p mimic), antagomiR-150–5p (anti-miR-150-5p) and their corresponding negative control (agomiR-NC, antagomiR-NC) were obtained from GenePharma (Shanghai, China), and their sequences were listed in Supplementary materials and methods. The agomiR-150-5p and agomiR-NC, antagomiR-150-5p and antagomiR-NC, siSP1, siSP2 and scramble, pcDNA3.1-VEGFA, pcDNA3.1-ZFAS1, pcDNA3.1-SP1, and blank vector (vector) were transfected into cells using Lipofectamine^TM^ 2000 (Invitrogen, USA) following the manufacturer’s protocol. Additionally, cell without transfection were treated with a VEGFR2 inhibitor Ki8751 (40 nM, Medchem Express, USA).

### Isolation of cytoplasmic and nuclear RNA

The distribution of ZFAS1 in the cytoplasmic and nuclear fractions of HCT116 and HCT8 cells were detected with the PARIS Kit (Life Technologies, USA) according to manufactures’ instructions.

### RNA in situ hybridization

In situ hybridization was used with a Fluorescent In Situ Hybridization Kit (RiboBio, China). Briefly, cells were plated on cover slips in a 24-well plate, and were fixed with 4% paraformaldehyde after three washes using 1 × PBS. Then cells were added 1 ml cold PBS containing 0.5% Triton X-100 at 4 ℃ for 5 min, washed with 1 × PBS three times, and prehybridizated at 37 ℃ for 0.5 h before hybridization. An anti-ZFAS1, anti-18S and anti-U6 oligodeoxynucleotide probe was performed in the hybridization solution at 37 ℃ overnight. Cells were counterstained with 4′,6-diamidino-2-phenylindole (DAPI, RiboBio, China) the following day and images were captured by fluorescence microscope (Nikon, Japan).

### Dual-luciferase reporter assays

The SP1-binding site in the promoter of ZFAS1 was identified using JASPAR database. The different fragment sequences of the promoter of ZFAS1 were synthesized and inserted into the pGL3-basic vector, and then cotransfected with SP1 expression plasmid into 293T cells.

293T and HCT116 cells were cotransfected with pmirGLO-ZFAS1-WT, pmirGLO-ZFAS1-Mut, pmirGLO-VEGFA-3′UTR-WT, pmirGLO-VEGFA-3′UTR-Mut reporter plasmids and agomiR-NC, agomiR-150-5p. Twenty-four hours after transfection, dual-luciferase reporter assay system (Promega, Madison, WI, USA) was performed to measure the relative luciferase activity and normalized to renilla luciferase activity.

### Chromatin immunoprecipitation (ChIP) assays

The EZ-Magna ChIP kit (Millipore, Billerica, USA) was employed to conduct ChIP assays. Briefly, the CRC cells were fixed with 1% formaldehyde solution for 15 min and incubated with 125 nM glycine for 5 min. DNA fragments ranging from 200 to 300 bp were generated using sonication. Antibodies including anti-SP1 (#9389, Cell Signaling Technology, USA) and IgG were employed for each immunoprecipitation. qPCR was used to analyze the precipitated DNA.

### RNA immunoprecipitation (RIP) assays

RIP assay was performed with Magna RIP^TM^ RNA-Binding Protein Immunoprecipation Kit (Millipore, Billerica, USA) and AGO2 antibody (proteintech, 10686–1-AP, China) in accordance with the manufacturer’s protocol. HCT116 cells were transfected with agomiR-150-5p or agomiR-150-5p negative control (agomiR-NC), after 48 h, cells were performed RIP using AGO2 antibody and then relative ZFAS1 expression was analyzed by qRT-PCR. The RIP fraction Ct value was normalized to the input RNA fraction Ct value.

### Biotinylated RNA pull-down assay

The pull-down assay with biotinylated RNA was performed as described previously^20^. Briefly, for ZFAS1 pulled down miRNAs, the biotinylated-ZFAS1 probe was incubated with C-1 magnetic beads (Life Technology) to generate probe-coated beads, incubated with sonicated CRC cells at 4 ℃ overnight, followed by eluted and qRT-PCR. For miR-150-5p pulled down ZFAS1, CRC cells with ZFAS1 overexpression were transfected with biotinylated miR-150-5p mimics or mutant using Lipofectamine 2000. Then the cells were harvested, lysed, sonicated, and incubated with C-1 magnetic beads (Life Technologies), followed by washed and qRT-PCR.

### Cell proliferation, colony formation, wound-healing, transwell, and tube formation assays

Detailed informations of cell proliferation, colony formation, wound-healing, transwell, and tube formation assays are described in Supplementary materials and methods. All experiments were performed more than three times.

### Western blotting

Detailed informations of western blotting were described in Supplementary materials and methods. Each experiment was performed more than three times.

### RNA isolation and quantitative RT-PCR

Detailed informations of RNA isolation and quantitative RT-PCR are described in Supplementary materials and methods.

### Tumor xenografts, tail vein injection and chicken chorioallantoic membrane (CAM) experiments

All animal experiments were approved by the animal care Committee of Nanjing First Hospital, Nanjing Medial University (acceptance No. SYXK 20160006). More detailed information of in vivo experiments are described in Supplementary materials and methods.

### Statistical analysis

All experiments were performed in triplicate. The data are expressed as mean ± SD, and analyzed by SPSS 22.0 software. The Student’s *t*-test, one-way analysis of variance (ANOVA) were used to estimate the differences between groups. Pearson’s Mann–Whitney *U* test or *χ*2 test was performed to analyze the relationship between expression of ZFAS1 and clinicopathological features. Kaplan-Meier method was applied to assess OS. The survival curves were compared with log-rank test. Follow-up time was censored if the patient was lost to follow-up. Cox proportional hazards model was used to perform multivariate analysis and calculate the 95% confidence interval (95% CI). *P* < 0.05 was considered to be statistically significant.

## Results

### Identification of lncRNAs that were differentially expressed in CRC tissues and ANTs in TCGA database

**Fig. 1:**
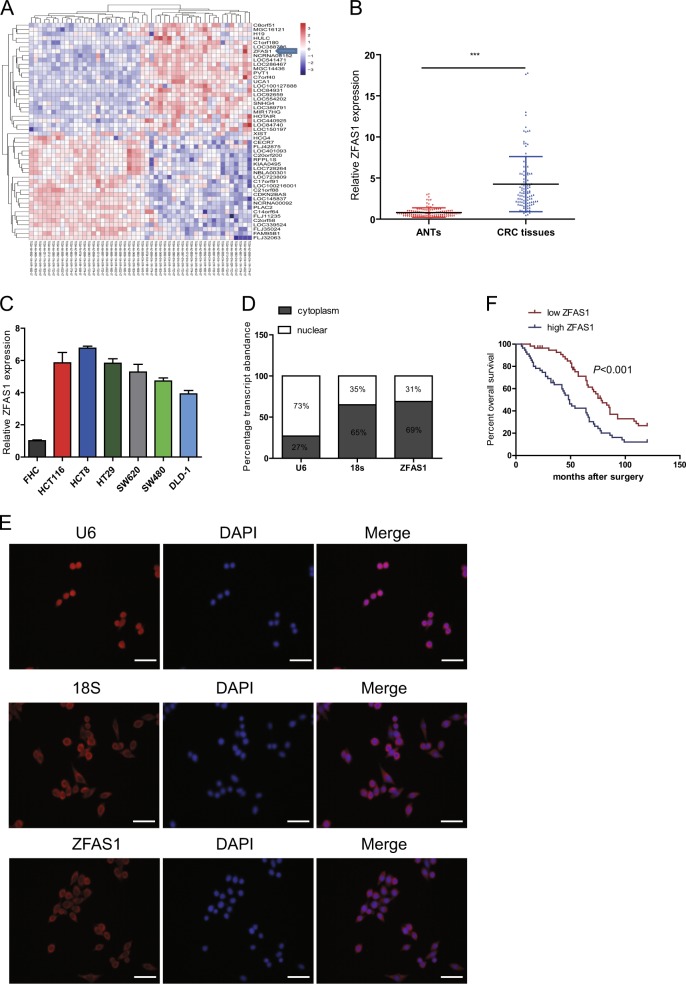
LncRNA-ZFAS1 was overexpressed in CRC tissues and cells, and is associated with poor overall survival. **a** A heat map revealed the differentially expressed lncRNAs between 25 CRC samples and corresponding adjacent normal tissues from TCGA database. **b** qRT-PCR analysis of ZFAS1 expression in 112 paired CRC tissues and corresponding adjacent normal tissues. GAPDH was used as an internal control. **c** qRT-PCR analysis of ZFAS1 expression in HCT116, HCT8, HT29, SW620, SW480, DLD-1, and FHC cells. **d** Nuclear and cytoplasmic RNA fractions were isolated from HCT116 cells. ZFAS1 was located mainly in the cytoplasm, 18S and U6 were used as controls. **e** RNA-FISH were performed to further verified that ZFAS1 was located mainly in the cytoplasm, 18S and U6 were used as controls. **f** Association of ZFAS1 expression with OS (Kaplan-Meier plot). ***P < 0.001

Sequencing data downloaded from the The Cancer Genome Atlas (TCGA) database revealed that dysregulated lncRNAs expression in 24 paires of colon adenocarcinoma tissues in comparison with the corresponding ANTs, ZFAS1 was marked with an arrow (Fig. 1a).

### ZFAS1 is upregulated in CRC tissues and cell lines, and is mainly located in the cell cell cytoplasm

QRT-PCR was performed to investigate the expression of ZFAS1 in CRC tissues and corresponding ANTs and found that ZFAS1 was markedly increased in CRC tissues compared with ANTs (Fig. 1b). High levels of ZFAS1 were also detected in HCT116, HCT8, HT29, SW620, SW480, and DLD-1 cells as compared with that in FHC cells, HCT116, and HCT8 were chosen for further experiments (Fig. 1c).

To detect the subcellular location of ZFAS1, qRT-PCR and fluorescence in situ hybridization (FISH) results both indicated that lncRNA-ZFAS1 mainly located in the cytoplasm (Fig. 1d, e).

### Correlation between ZFAS1 expression and clinicopathological characteristics and prognosis of CRC

To explore the clinical relevance of ZFAS1 expression, we divided CRC patients into two groups. Its expression was markedly correlated with TNM stage, pT stage, and distant metastasis (Table 1). The results indicated that high expression of ZFAS1 is associated with malignant progression in CRC patients.

We performed Meier-Kaplan to evaluate the prognostic value of ZFAS1 in CRC, the data showed that patients with high ZFAS1 had a poorer OS, when compared to patients with low ZFAS1 (Fig. 1f). Additionally, Cox regression analyses were performed to further assess the prognostic value of ZFAS1 in CRC, multivariate survival analyses indicated that ZFAS1 expression was strongly associated with OS, moreover, univariate analyses showed that ZFAS1 could be regarded as an independent predictor for OS (Table 2).

**Table 2:** univariate and multivariate analysis for OS in patients with CRC

Characteristics	Multivariate analysis for OS		Univariate analysis for OS	
	HR (95% CI)	*P*	HR (95% CI)	*P*
Gender (male/female)	–	–	1.158 (0.739–1.812)	0.522
Age (<60/ ≥ 60)	–	–	0.928 (0.618–1.551)	0.928
Tumor location (colon/rectal)	–	–	1.160 (0.754–1.785)	0.500
TNM stage (I–II/III–IV)	2.012 (1.134–3.423)	0.006	2.073 (1.339–3.209)	0.001
Differentiation (low/moderate/high)	–	–	1.322 (0.935–1.871)	0.114
PT stage (T1–T2/T3–T4)	–	–	0.674 (0.357–1.271)	0.223
PN stage (N0/N1/N2)	1.624 (1.013–2.871)	0.001	1.814 (1.182–2.785)	0.006
Distant metastasis (M0/M1)	3.012 (1.493–5.834)	< 0.001	2.702 (1.451–5.031)	0.002
Tumor size (<5 cm/ ≥ 5 cm)	–	–	0.654 (0.420–1.018)	0.060
CEA (<10 ng/ml/ ≥ 10 ng/ml)	–	–	0.775 (0.479–1.192)	0.228
CA199 (<10U/ml/ ≥ 10U/ml)	–	–	0.751 (0.488–1.156)	0.193
ZFAS1 (low/high)	1.474 (1.121–2.573)	0.013	2.045 (1.321–3.166)	0.001

*CI* confidence interval, *HR* hazard ratio

### SP1 activated ZFAS1 expression in the CRC cells

**Fig. 2:**
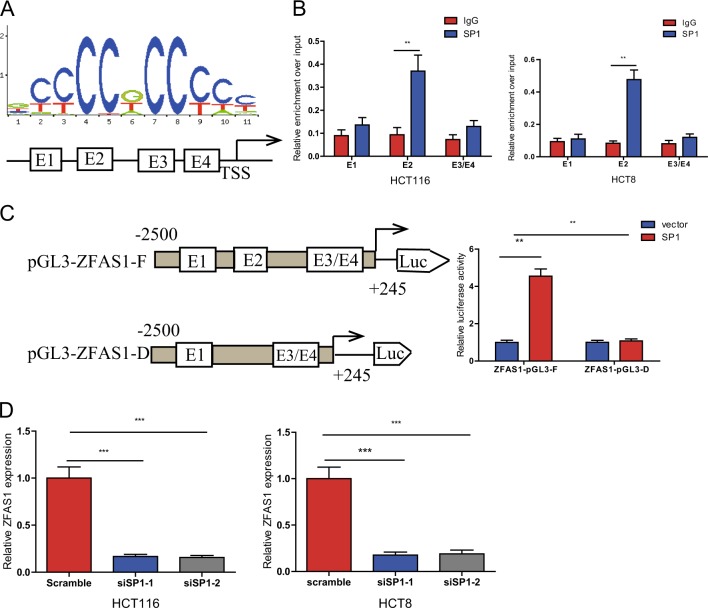
The transcription factor SP1 is involved in ZFAS1 upregulation. **a** The predicted positions of puative SP1 binding motif in −2500 bp human ZFAS1 promoter. **b** Quantitative ChIP assays were performed to show direct binding of SP1 to endogenous ZFAS1 promoter regions. The primers designed for ChIP were provided in supplementary materials and methods. **c** A luciferase reporter assay was used by cotransfecting the full ZFAS1 promoter (ZFAS1-pGL3-F) or deleted ZFAS1 promoter fragment E2 (ZFAS1-pGL3-D) with SP1 expression plasmid or blank vector in 293T cells. Luciferase activities were expressed as relative to that of the pGL3 vector. **d** qPCR analysis of ZFAS1 expression levels following the treatment of siSP1-1, siSP1-2 in HCT116 and HCT8 cells. Data were shown as mean ± SD of three independent experiments. **P < 0.01, ***P < 0.001

Accumulating evidences have been reported that several epigenetic regulators and key transcription factors (TFs) contributed to lncRNAs dysregulation in human cancers, such as Stat3^21^, p53^22^, EZH2^23^. Although we have found ZFAS1 was overexpressed in CRC tissues and cell lines, the factors involved in ZFAS1 dysregulation remained elusive. Using JASPAR database (http://jaspardev.genereg.net/), we found that there are four SP1 binding sites with scores >10 at the regions E1 (−1330 to −1320, TCTCCTCCTCT), E2 (−766 to −756, GCTCCGCCCAG), E3 (−44 to −34, CCCCACCCCG) and E4 (−19 to −9, TCCCCTCCCTC) in the ZFAS1 promoter regions (Fig. 2a).

Firstly, we designed three paired primers and ChIP assay was performed to detect which region in the ZFAS1 promoter mediated SP1-binding to the ZFAS1 promoter. The ChIP data showed that SP1 could bind to E2 sites (Fig. 2b). To further verify this result, we cloned the full promoter region of ZFAS1 and E2 deleted promoter region into pGL3-basic reporter. The results showed that the deletion of E2 sites significantly impaired the effect of SP1 on ZFAS1 transcription activation (Fig. 2c), revealing that SP1 could bind to the promoter of ZFAS1 to regulate ZFAS1 transcription.

Moreover, we explored whether the overexpression of ZFAS1 is mediated by SP1. We silenced endogenous SP1 expression in CRC cells by transfecting with siRNAs (siSP1–1, siSP-2) targeting the SP1 gene. ZFAS1 levels were markedly decreased in CRC cells transfected with siRNAs (Fig. 2d). Furthermore, we detected the expression of SP1 mRNA in CRC cell lines and tissues and found that SP1 mRNA was markedly upregulated in CRC cells and tissues (Fig S1A, S1B). We also found that SP1 mRNA and ZFAS1 expression levels have positive correlation (Fig S1C). These results indicated that ZFAS1 upregulation in CRC is mediated by SP1.

### ZFAS1 overexpression promoted CRC cells growth, migration, invasion, and HUVECs tube formation

**Fig. 3:**
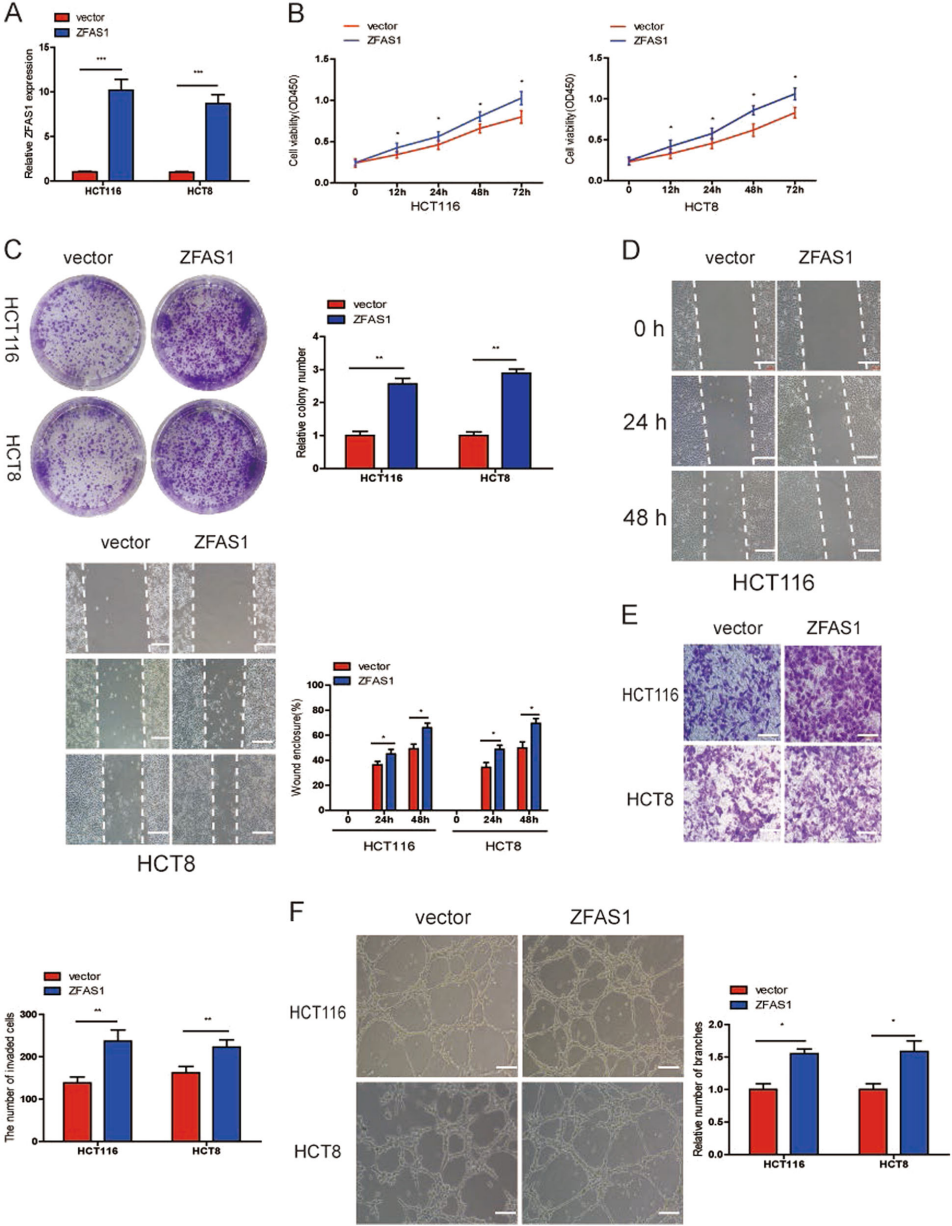
ZFAS1 upregulation promoted CRC cells growth, migration, invasion, and HUVECs tube formation. **a** Relative ZFAS1 expression was assessed after transfection with pcDNA3.1-ZFAS1 (ZFAS1) or blank vector (vector). **b**, **c** The proliferation of HCT116 and HCT8 cells transfected with ZFAS1 or blank vector were measured using CCK-8 (**b**) and colony formation (**c**). **d**, **e** Wound healing (**d**) and transwell invasion (**e**) assays were performed to evaluate the ability of migration and invasion in HCT116 and HCT8 cells transfected with ZFAS1 or blank vector, respectively. **f** HUVECs were cultured in TCM derived from HCT116 and HCT8 cells transfected with pcDNA3.1-ZFAS1 or blank vector, the relative number of tube branches were measured in random 8 photographic fields. Each experiment were performed three times. Data were shown as mean ± SD. *P < 0.05, **P < 0.01

To detect the biological function of ZFAS1 in CRC, we selected HCT116 and HCT8 cells for pcDNA-3.1-ZFAS1 or blank vector transfection. The efficiency of transfection was confirmed by qRT-PCR (Fig. 3a). Firstly, CCK-8 assay was performed to assess CRC cell growth and found that upregulation of ZFAS1 obviously increased cell proliferation (Fig. 3b), a colony formation assay was further substantiated the role of ZFAS1 in promoting proliferation of CRC cells (Fig. 3c). Next, wound-healing and transwell assays were performed in HCT116 and HCT8 cells, as shown in Fig. 3d, e, the migratory and invasive ability were significantly elevated when the expression of ZFAS1 was increased. Additionally, to explore whether ZFAS1 is a key angiogenesis promoter in CRC, we investigated the influence of ZFAS1 on HUVECs tube formation, and the results showed that ZFAS1 upregulation markedly increased the ability of HUVECs tube-formation (Fig. 3f).

### Knockdown of ZFAS1 lead to an obvious inhibition of proliferation, migration, invasion, and angiogenesis in vitro

**Fig. 4:**
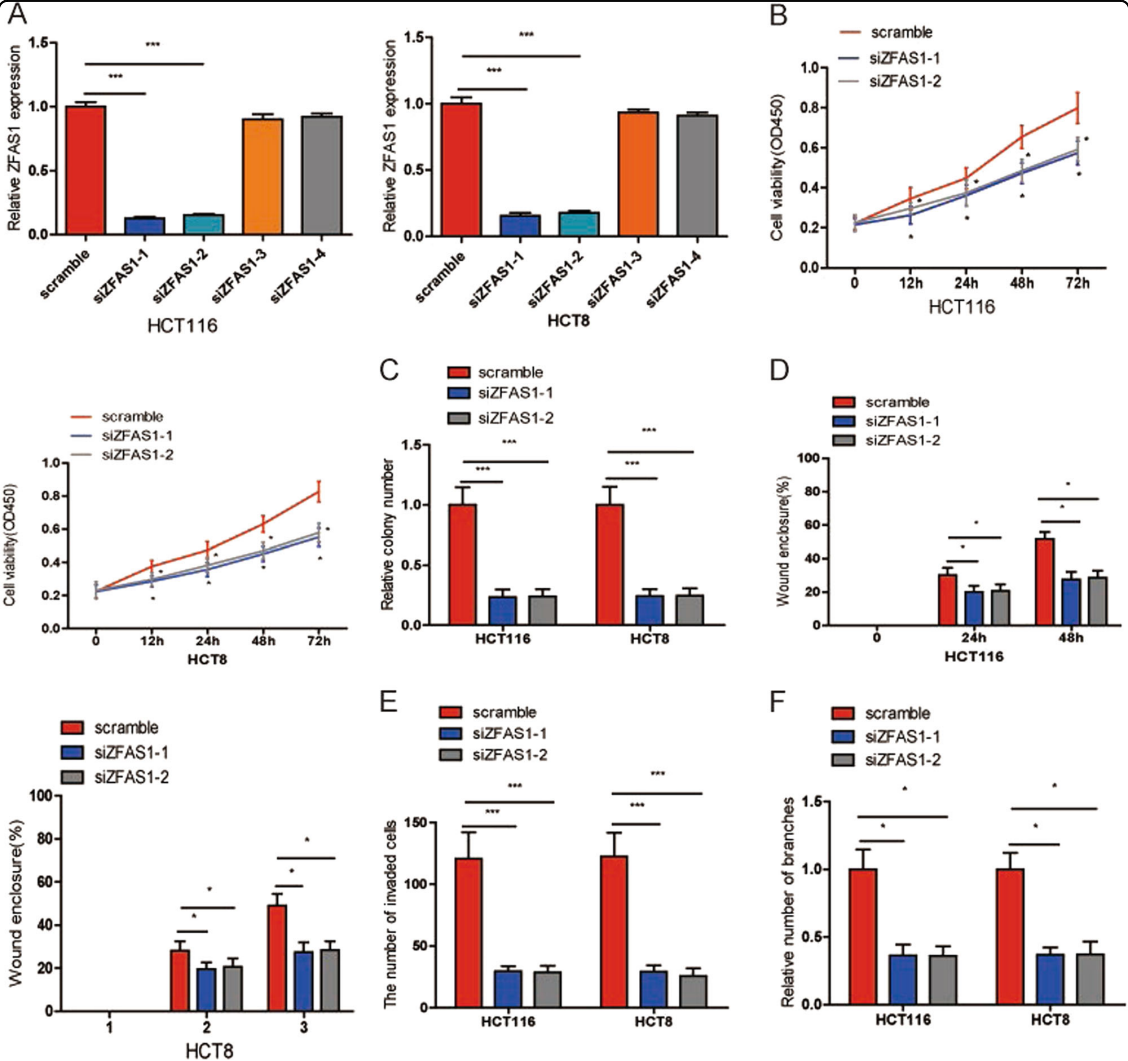
ZFAS1 knockdown inhibited CRC cells proliferation, migration, invasion, and HUVECs tube formation. **a** The relative expression of ZFAS1 were detected in HCT116 and HCT8 cells after transfecting with siZFAS1-1, siZFAS1-2, siZFAS1-3, siZFAS1-4 or scramble. **b**, **c** Cells proliferation were assessed in ZFAS1 knockdown HCT116 and HCT8 cells using CCK-8 (**b**) and colony formation (**c**). **d**, **e**. Wound healing (**d**) and transwell invasion (**e**) assays were used to evaluate the ability of ZFAS1 knockdown CRC cells. **f** HUVECs were cultured in TCM derived from ZFAS1 knockdown HCT116 and HCT8 cells. The relative number of tube branches were measured in random 8 photographic fields. Each experiment were performed three times. Data were shown as mean ± SD. *P < 0.05, ***P < 0.001

Next, we also knocked down ZFAS1 expression using siRNA in HCT116 and HCT8 cell lines. QRT-PCR showed that si-ZFAS1–1 and siZFAS1-2 were both significantly downregulated in both HCT116 and HCT8 cells compared to cells with scramble (Fig. 4a). Then we constructed four stable knockdown CRC cells (HCT116 and HCT8) using ZFAS1 siRNA lentivirus (siZFAS1-1, siZFAS1–2) and two control CRC cells (HCT116 and HCT8) using a scramble siRNA GFP Lentivector (scramble) via treatment of puromycin. We found that inhibition of ZFAS1 could markedly suppressed CRC cells proliferation (Fig. 4b, c and S2A), migration (Fig. 4d and S2B), invasion (Fig. 4e and S2C) and HUVECs tube formation (Fig. 4f and S2D).

### ZFAS1 directly interacts with miR-150-5p

Many lncRNAs have been reported to act as competing endogenous RNAs (ceRNAs).

**Fig. 5:**
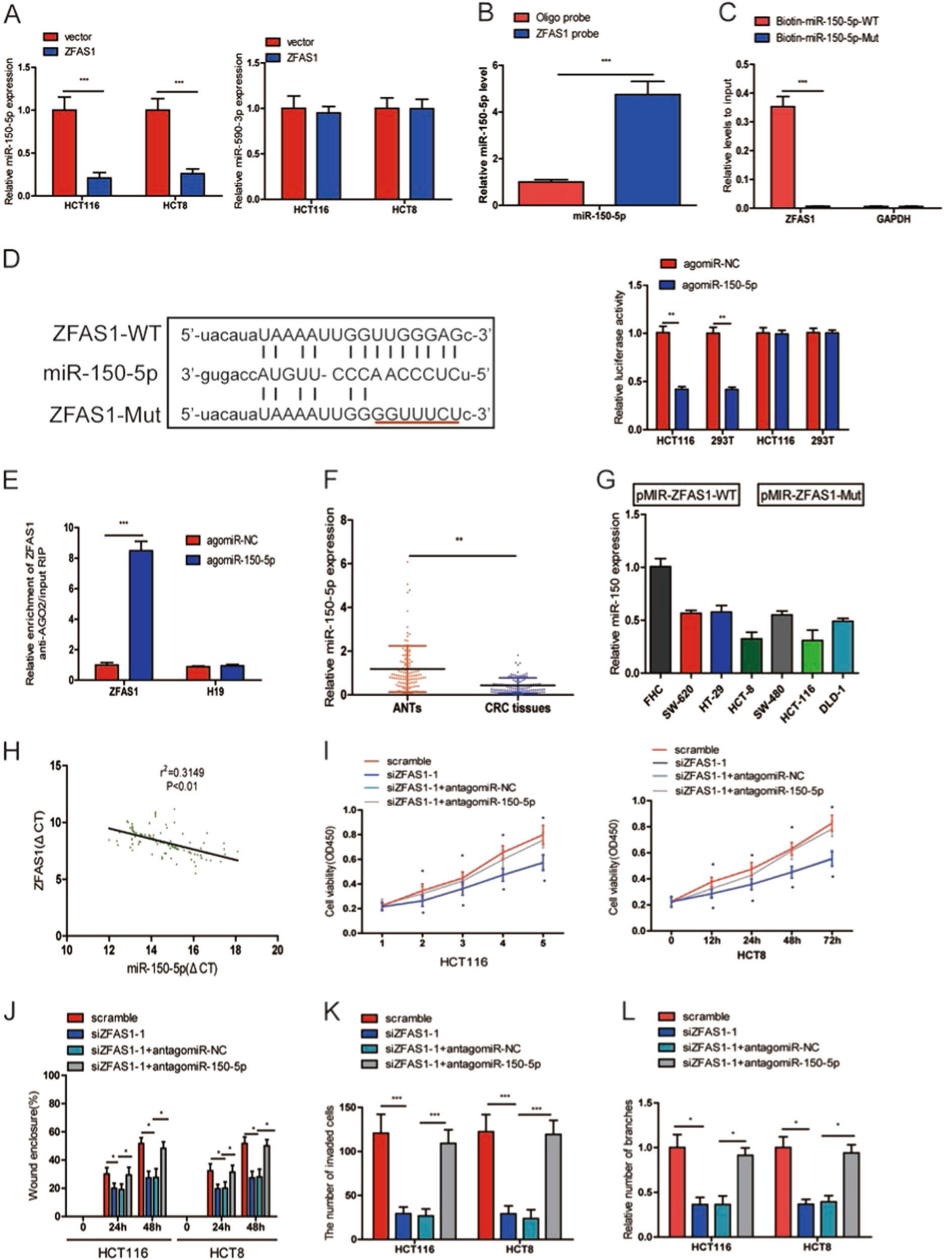
ZFAS1 functioned as a competing endogenous RNA (ceRNA) by sponging miR-150-5p. **a** The expression of miR-150-5p and miR-590–3p were detected in HCT116 and HCT8 cells after transfecting with pcDNA3.1-ZFAS1 or blank vector. **b** The expression of miR-150-5p was detected using qRT-PCR after the biotinylated-ZFAS1 pull down assay in HCT116 cells. **c** The biotinylated wild-type/mutant miR-150-5p was transfected into HCT116 cells with ZFAS1 overexpression. The expression levels of ZFAS1 were measured by qRT-PCR after streptavidin capture. **d** Wild and mutant ZFAS1 sequences were cloned into pmirGLO reporter, Luciferase activity in HCT116 and 293T cells cotransfected with agomiR-150-5p or agomiR-NC and pmirGLO-ZFAS1-WT or pmirGLO-ZFAS1-Mut. Luciferase activities were normalized to renilla luciferase. **e** Anti-AGO2 RIP was used in HCT116 cells overexpressing agomiR-150-5p, followed by qRT-PCR to evaluate the expression of ZFAS1 or H19 (control) associated with AGO2. The data are shown as the mean ± SD of three independent experiments. **f** miR-15-5p was downregulated in CRC tissues compared to paired adjacent normal tissues. **g** The expression of miR-150-5p in HCT116, HCT8, HT29, SW620, SW480, DLD-1, and FHC. **h** The correlation between ZFAS1 level and miR-150-5p level in CRC tissues. **i** CCK-8 proliferation assays in siZFAS1-1 and antagomiR-150-5p transfected HCT116 and HCT8 cells. **j** Wound healing assays in siZFAS1-1 and antagomiR-150-5p transfected HCT116 and HCT8 cells. **k** Transwell invasion assays in siZFAS1-1 and antagomiR-150-5p transfected HCT116 and HCT8 cells. **l** HUVECs tube formation in siZFAS1-1 and antagomiR-150-5p transfected HCT116 and HCT8 cells. Each experiment were performed three times. Data were shown as mean ± SD. *P < 0.05, **P < 0.01, ***P < 0.001

Using starbase (http://starbase.sysu.edu.cn/), we predicted that ZFAS1 could be targeted by miR-150-5p and miR-590-3p. To verify which could bind to ZFAS1, we detected the expression of miR-150-5p and miR-590-3p in HCT116 and HCT8 cells after transfecting with pcDNA3.1-ZFAS1. The results showed that miR-150-5p expression levels was markedly decreased while miR-590-5p expression levels did not alter after ZFAS1 upregulation (Fig. 5a). Then, RNA pull down assay showed that miR-150-5p was abundantly pulled down by ZFAS1 probe in comparison with oligonucleotide probe in HCT116 cells (Fig. 5b). To further verify the interaction between them, ZFAS1- overexpressing cells were transfected with wild-type or mutant biotinylated miR-150-5p mimics, and the data suggested that wild-type miR-150-5p mimics captured more ZFAS1 than mutant miR-150-5p mimics (Fig. 5c).

To further prove that ZFAS1 could bind to miR-150-5p, the wild-type sequence of ZFAS1 (ZFAS1-WT) or its mutant sequence (ZFAS1-Mut) was cloned into the pmirGLO luciferase reporter and agomiR-150-5p or agomiR-NC, agomiR-150-5p significantly reduced luciferase activity of the pmirGLO-ZFAS1-WT vector, but failed to decrease luciferase activity of pmirGLO-ZFAS1-Mut vector. We preliminary showed that ZFAS1 was a target of miR-150-5p (Fig. 5d).

miRNAs bind their target genes and exert the ability of translational repression or RNA degradation mainly in an AGO2-dependent manner. To further explore whether ZFAS1 binds to miR-150-5p in this manner. RIP was used in HCT116 and confirmed the interaction between ZFAS1 and miR-150-5p (Fig. 5e). These results strongly supported the idea that ZFAS1 acted as a miR-150-5p sponge in CRC.

### MiR-150–5p was downregulated in CRC and negatively correlated with ZFAS1

We detected miR-150-5p expression using qRT-PCR in CRC tissues and cell lines. miR-150-5p was reduced in CRC tissues compared with corresponding ANTs (Fig. 5f). As shown in Fig. 5e, miR-150-5p expression in HCT116, HCT8, HT29, SW620, SW480, and DLD-1 was markedly downregulated in comparison with FHC (Fig. 5g). Moreover, ZFAS1 levels were significantly negatively correlated with miR-150-5p in CRC tissues (Fig. 5h). Additionally, we found that decreased ability of CRC cells proliferation, migration, invasion and HUVECs tube formation caused by ZFAS1 knockdown could be reversed by inhibition of miR-150-5p (Fig. 5i, j and S3A, 5k and S3B, 5L and S3C).

### ZFAS1 knockdown inhibited the progression and development in CRC in vivo

**Fig. 6:**
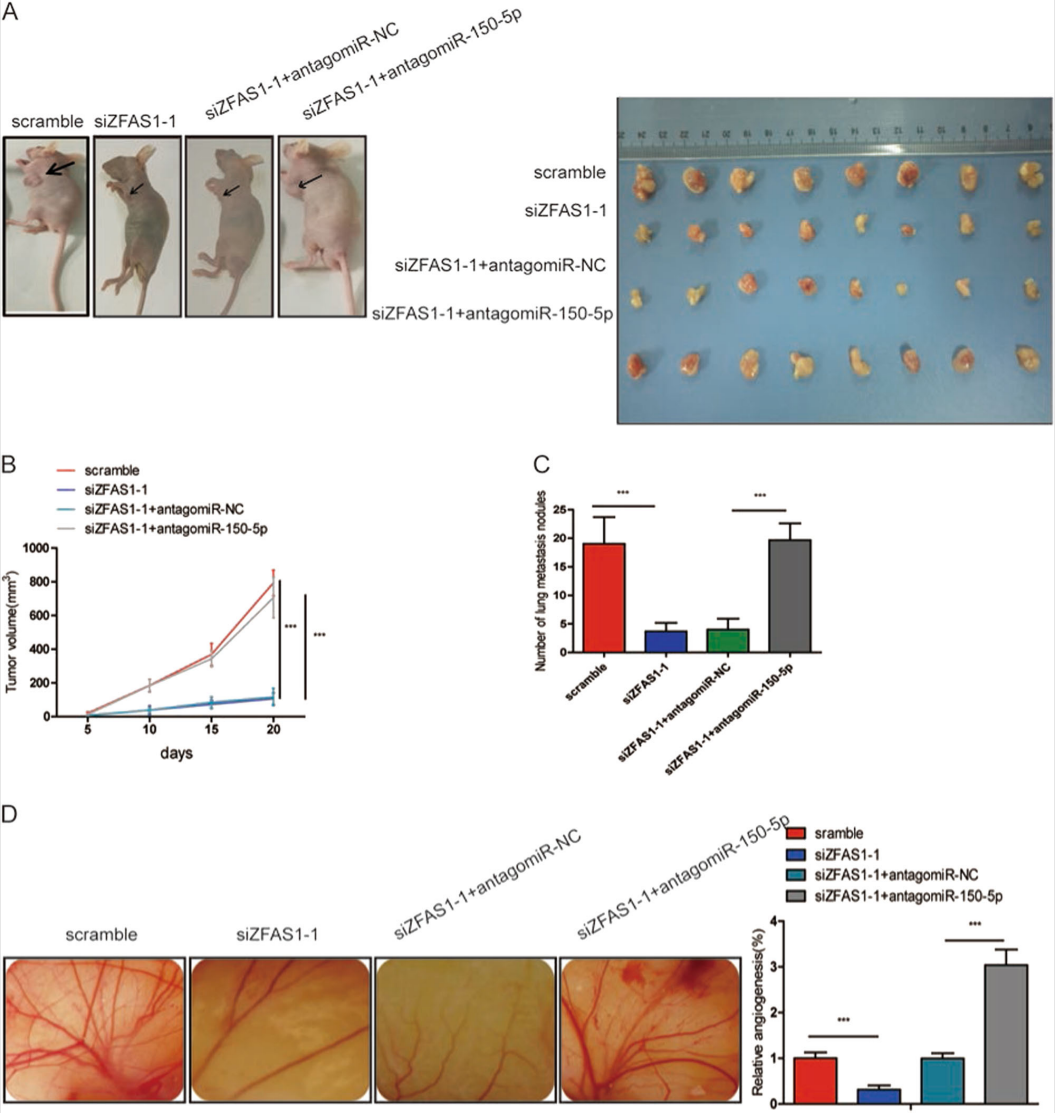
ZFAS1 knockdown inhibited tumor growth, metastasis, and angiogenesis in vivo. **a**, **b** Subcutaneous implant model was established using HCT116 cells. The volume of xenograft tumors in four groups (*n* = 8). Data are presented as the mean ± SD. **c** The number of metastatic nodules in the lungs of mice (three sections evaluated per lung) from four groups (*n* = 8). **d** Chicken embryos were incubated with conditioned medium (CM) from the four groups (*n* = 8) for 4 days, then photographed with a camera and quantified by vessel count. Each experiment were performed three times. Results are presented as mean ± SD. ***P < 0.001

To test the biological function of ZFAS1 in vivo, different HCT116 cells were injected into nude mice subcutaneously or intravenously. These mice were divided into four groups: group 1 (scramble) was injected with scramble HCT116 cells; group 2 (siZFAS1-1) was injected with ZFAS1 knockdown HCT116 cells; group 3 (siZFAS1-1 + antagomiR-NC) was injected with ZFAS1 knockdown HCT116 cells transfected with antagomiR-NC; group 4 (siZFAS1-1 + antagomiR-150-5p) was injected with ZFAS1 knockdown HCT116 cells transfected with antagomiR-150-5p. We found that the volume of tumors in the siZFAS1-1 were significantly smaller than that in the scramble group, and that antagomiR-150-5p could partially increase the growth trend caused by ZFAS1 knockdown (Fig. 6a, b).

To evaluate the influence ZFAS1 had on metastasis in vivo. The same four groups of cells clone were injected into mice through tail vein. After 8 weeks of injection, CT scans were used on each mouse and the results showed that the number of metastatic nodules in siZFAS1-1 group was obvious less than that in scramble group, while inhibition of miR-150-5p could partially abrogate the inhibition of lung metastasis caused by siZFAS1-1 (Fig S4). Following CT scanning, mice were sacrificed and HE were performed on each lung. The number of lung metastatic nodules counted under the microscope in each group was consistent with CT scan results (Fig. 6c and S4).

Then CAM assay was utilized to further exam the effect of ZFAS1 on CRC angiogenesis in vivo, compared with scramble group, siZFAS1-1 group had a significantly reduced number of new blood vessels formation, while antagomiR-150-5p showed a significant increase number of blood vessels formation (Fig. 6d). These data further demonstrated that ZFAS1, acting as a miR-150–5p sponge, promoted CRC progression.

### VEGFA is a direct target gene of miR-150-5p

**Fig. 7:**
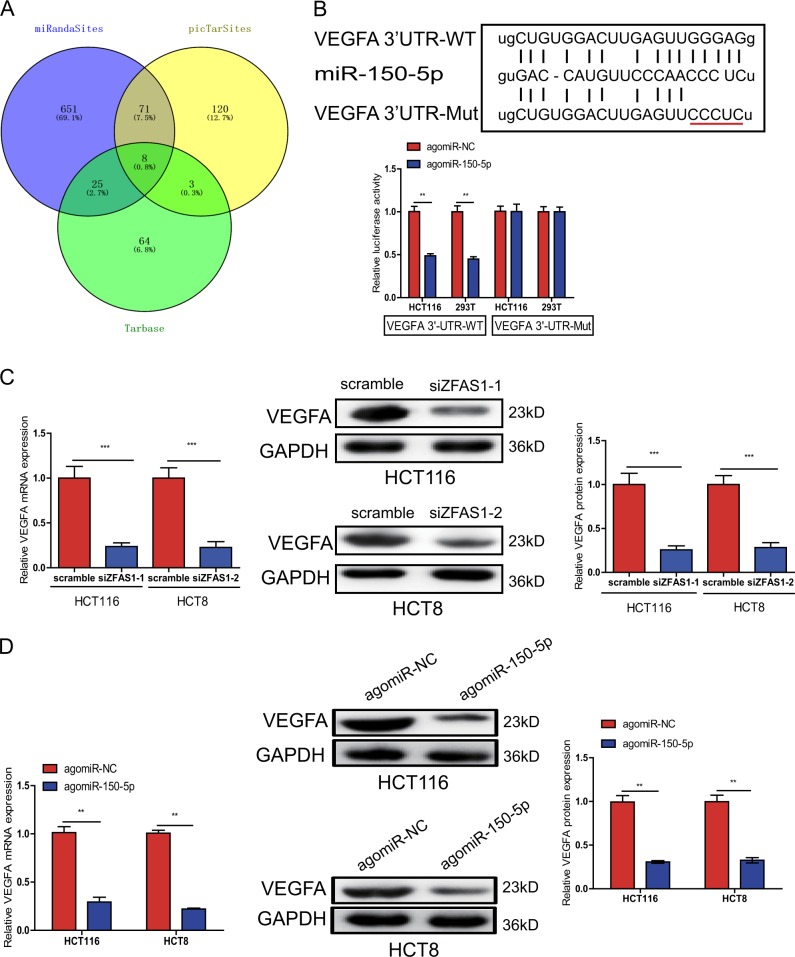
VEGFA was identified as a direct target of miR-150-5p in CRC cells. **a** The direct target genes of miR-150-5p were predicted using PicTarSites, miRandaSites, and Tarbase databases. **b** Wild and mutant VEGFA-3′UTR sequences were cloned into luciferase reporter, Luciferase activity in HCT116 and 293T cells cotransfected with agomiR-150-5p or agomiR-NC and pmirGLO-VEGFA-3′UTR-WT or pmirGLO-VEGFA-3′UTR-Mut. Luciferase activities were normalized to renilla luciferase. **c**, **d** QRT-PCR and Western blot analysis showed that both VEGFA mRNA and protein expression levels were dramatically suppressed by siZFAS1(**c**) or agomiR-150-5p (**d**) in HCT116 and HCT8 cells. **P < 0.01, ***P < 0.001

Mounting miRNAs have been reported to be dysregulated and exert their functions mainly through their downstream target genes. PicTarSites, miRandaSites, and Tarbase databases were performed to search for potential target genes of miR-150-5p. As shown in Fig. 7a, eight potential genes were predicted in all four database. VEGFA which was selected as a target gene of miR-150-5p have been reported to be closely associated with metastasis and angiogenesis of human cancers. Next, dual-luciferase reporter assays were used to determine whether VEGFA is a direct target gene in CRC cells. 3′-UTR of VEGFA wild-type (WT-VEGFA-3′UTR) as well as VEGFA mutant type (Mut-VEGFA-3′UTR) were constructed and cloned into pmirGLO luciferase reporter, then cotranfected with agomiR-150-5p or agomiR-NC into HCT116 and 293T cells. Luciferase reporter assay confirmed that miR-150-5p overexpression obviously reduced the luciferase activity of pmirGLO-VEGFA-3′UTR-WT, but showed no significantly effect on the luciferase activity of pmirGLO-VEGFA-3′UTR-Mut (Fig. 7b).

Additionally, we found that either ZFAS1 knockdown or miR-150-5p overexpression could dramatically decreased VEGFA expression on mRNA and protein levels in HCT116 and HCT8 cells using qRT-PCR and western blot analysis (Fig. 7c, d).

### ZFAS1 knockdown inhibited CRC progression via inhibiting miR-150-5p-mediated VEGFA/VEGFR2/Akt/mTOR signaling and EMT process

**Fig. 8:**
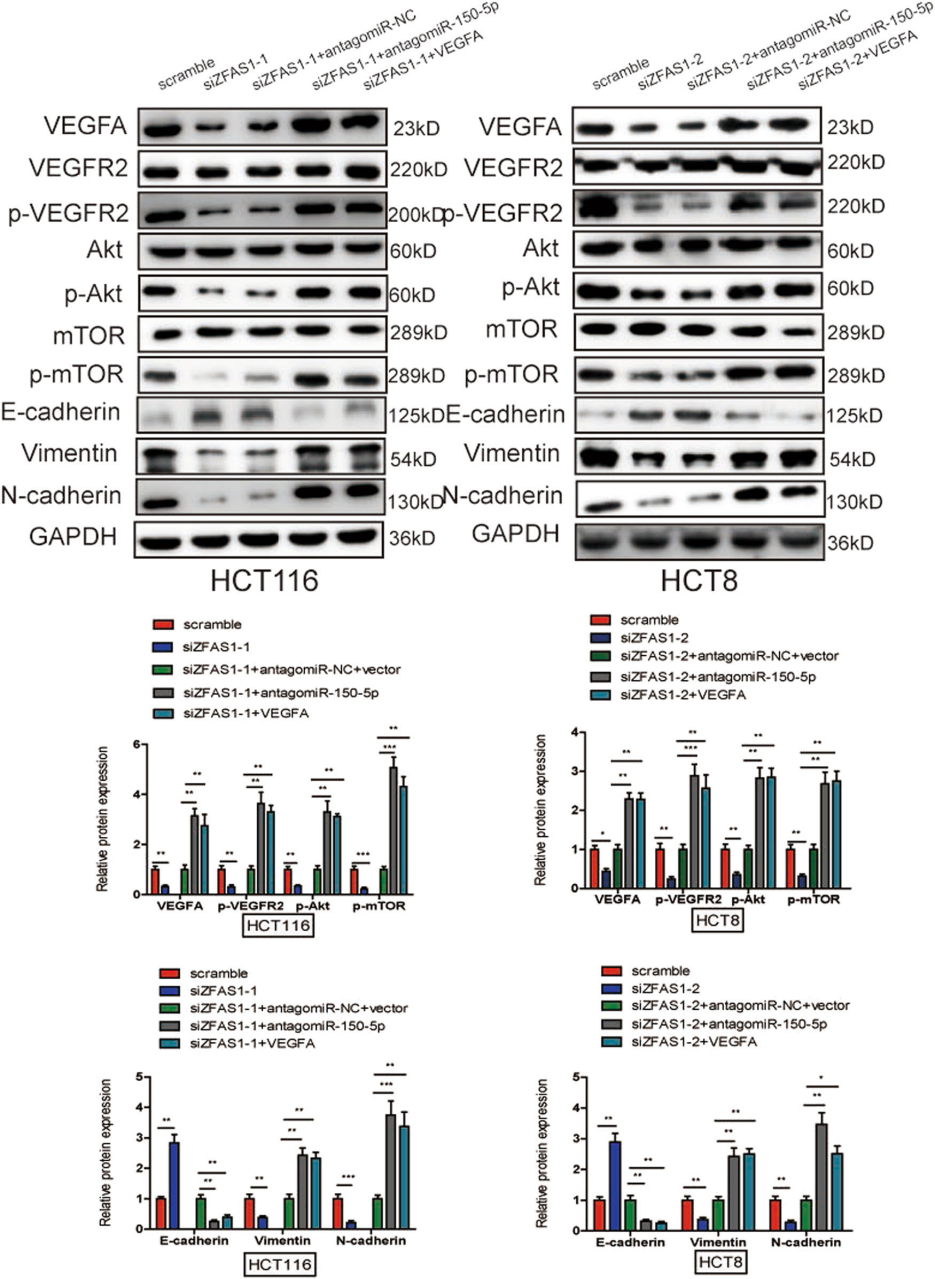
ZFAS1 knockdown inhibited CRC progression via inhibiting EMT process and inactivating Akt/mTOR signaling pathway. Western blot was used to measure the expression of VEGFA, VEGFR2, p-VEGFR2, Akt, p-Akt, mTOR, p-mTOR, E-cadherin, Vimentin, and N-cadherin after ZFAS1 silencing HCT116 and HCT8 cells transfected with antagomiR-150-5p and VEGFA or their negative control, GAPDH was performed as a loading control. Data were showed as mean ± SD of three independent experiments. *P < 0.05, **P < 0.01, ***P < 0.001

Overwhelming evidences had demonstrated that VEGFA could bind to VEGFR2, then activate PI3K and its downstream target Akt and mTOR which play key roles in cancer proliferation, metastasis, angiogenesis, and survival^24–26^. To further explore whether ZFAS1 knockdown inhibited CRC development through VEGFA-mediated Akt/mTOR signaling pathway. We found that ZFAS1 knockdown significantly decreased the level of VEGFA, and then not only significantly suppressed the phosphorylation of VEGFR2 (p-VEGFR2), Akt (p-Akt) and mTOR (p-mTOR), but also induce the changes of EMT-related markers (E-cadherin, Vimentin, N-cadherin) (Fig. 8). Next, we treated ZFAS1 knockdown HCT116 and HCT8 cells with antagomiR-150–5p and VEGFA, we found that antagomiR-150-5p and VEGFA treatment could at least partially increase the expression of p-VEGFR2, p-Akt, and p-mTOR and restore the hallmarks of the EMT (Fig. 8).

**Fig. 9:**
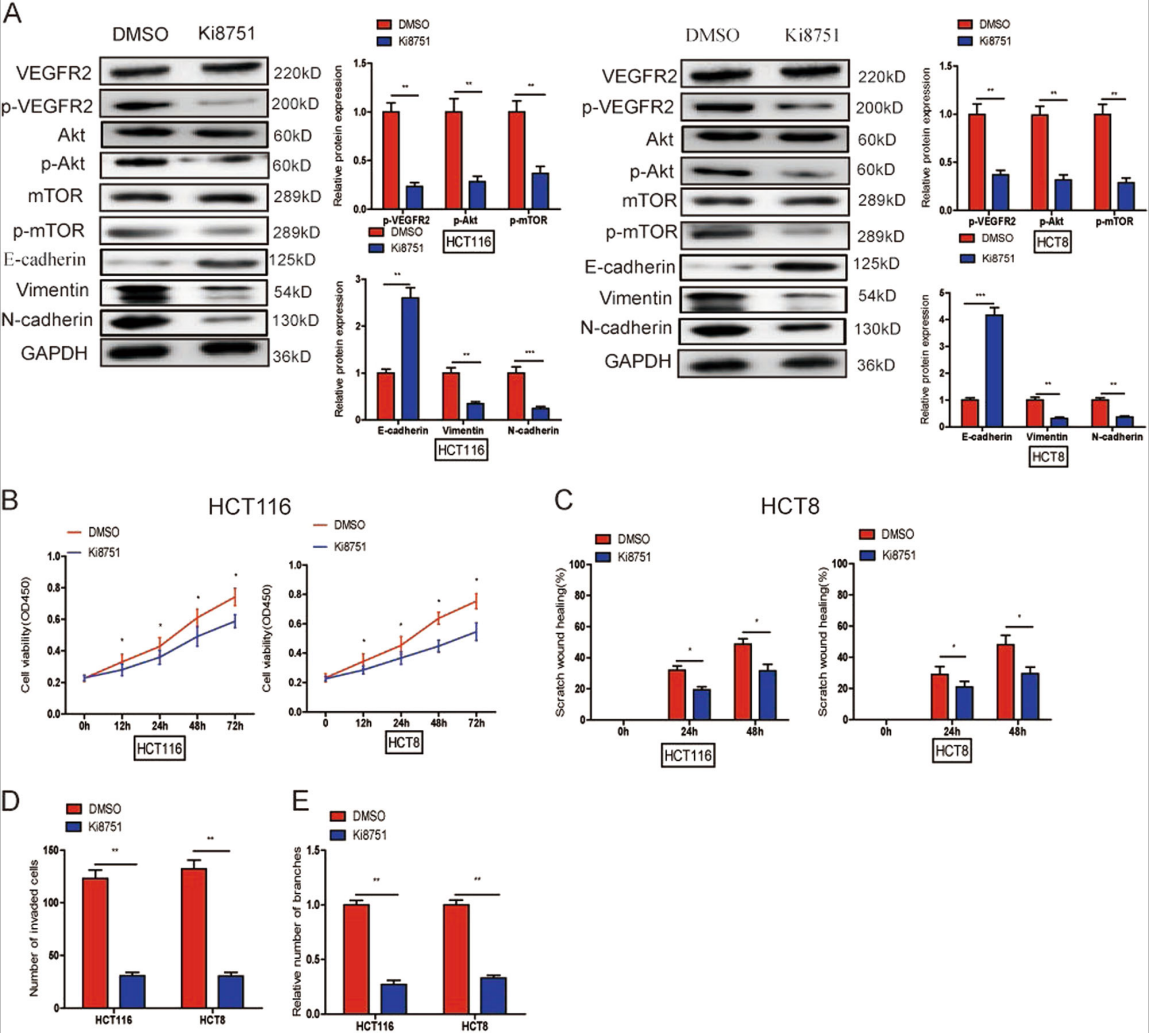
Ki8751 could suppress CRC progression through EMT process and inactivating Akt/mTOR signaling pathway. **a** Western blot was performed to detect the expression of VEGFR2, p-VEGFR2, Akt, p-Akt, mTOR, p-mTOR, E-cadherin, Vimentin, and N-cadherin after adding Ki8751 to HCT116, and HCT8 cells. **b**–**d** Ki8751 inhibited proliferation (**b**), migration (**c**), and invasion (**d**) of HCT116 and HCT8 cells, and suppressed HUVECs tube formation (**e**). Results are presented as mean ± SD. *P < 0.05, **P < 0.01, ***P < 0.001

Next, a specific VEGFR2 inhibitor Ki8751 to further explore whether ZFAS1 knockdown suppressed CRC progression via VEGFA/VEGFR2 inactivation. The results showed that Ki8751 markedly repressed the phosphorylation of VEGFR2, Akt, mTOR (Fig. 9a), and decreased the expression of Vimentin and N-cadherin as well as increased the expression of E-cadherin (Fig. 9a). Moreover, Ki8751 could also significantly suppressed CRC cells proliferation (Fig. 9b), migration (Fig. 9c, S5A), invasion (Fig. 9d, S5B) and HUVECs tube formation (Fig. 9e, S5C).

These results revealed that VEGFA/VEGFR2 and downstream Akt/mTOR signaling pathway activations are essential for ZFAS1 mediated oncogenic function.

## Discussion

Recently, lncRNAs have gained great attention as its role in carcinogenesis and cancer progression^6,27–29^, several known lncRNAs (SNHG5, CCAL, CASSC11) were reported to be dysregulated in CRC tissues and involved in the CRC development and progression^30–32^. In our study, many differential expressed lncRNAs were identified between CRC tissues and ANTs from TCGA database, of these genes, we selected ZFAS1 to further study in vitro and in vivo. We found ZFAS1 was upregulated in CRC cell lines and tissues, which was consistent with the results from TCGA database, moreover, ZFAS1 was found to be closely associated with OS, pT stage, distant metastasis, and TNM stage. Recently, accumulating data have showed that SP1 was a key transcription factor in controlling lncRNAs expression^33,34^. Following ChIP and luciferase reporter assays, both determined that SP1 could interact with the promoter of ZFAS1, these essential data revealed that SP1 activated ZFAS1 translational expression to modulate ZFAS1 in CRC.

Functional investigations showed that ZFAS1 overexpression could significantly promoted, while ZFAS1 silencing inhibited CRC cells proliferation, migration, invasion, and HUVECs tube formation. These results indicated that ZFAS1 functioned as an oncogene in CRC.

Increasing studies have demonstrated that lncRNAs could regulate mRNA levels by competing for miRNAs, by acting as ceRNAs^35–37^. Lu et al.^12^ have reported that lncRNA BC032469 may function as sponge for miR-1207-5p to regulate hTERT expression. In this study, we showed ZFAS1 mainly located in the cytoplasm, bioinformatics analysis indicated that ZFAS1 could interact with miR-150-5p, RNA pull down, RIP and luciferase reporter assay were performed to further verify that ZFAS1 is able to bind to miR-150-5p. We also found that ZFAS1 expression levels were markedly reversely correlated with the expression of miR-150-5p in CRC tissues. Moreover, we verified that inhibition of miR-150-5p could abolished the ZFAS1 knockdown mediated biological effect in vitro and in vivo. Together, these data strongly supported the role of ZFAS1 as a miR-150-5p sponge in CRC.

miRNAs exert their function by modulating their target genes via elevating translational repression or mRNA degradation^38,39^. miR-150-5p have been reported be aberrant expressed in various malignancies, including non-small cell lung cancer (NSCLC), melanoma, Juvenile myelomonocytic leukemia (JMML)^40–42^. In our current study, we observed that miR-150-5p was downregulated in CRC cell lines and tissues. The mechanism of action of miR-150-5p was investigated via its target gene, we identified VEGFA was a target of miR-150-5p using multiple lines of evidences. Firstly, miR-150-5p was able to inhibit wild type of VEGFA-3′UTR activity in a luciferase reporter assay. Secondly, miR-150-5p overexpression reduced the expression of VEGFA both on mRNA and protein levels.

To better understand the underlying mechanism of ZFAS1 in CRC, on the one hand, VEGFA could bind to its receptors and induce an EMT, which is closely correlated with cancer metastasis and E-cadherin, Vimentin, and N-cadherin are important markers of EMT^43–45^. Our studies suggested that ZFAS1 knockdown significantly decreased the expression of Vimentin and N-cadherin, and increased the expression of E-cadherin. While antagomiR-150-5p and VEGFA could markedly upregulate Vimentin and N-cadherin, and downregulate E-cadherin. On the other hand, previous studies have reported that Akt/mTOR signaling pathway played a critical role in various biological processes of cancers, such as proliferation, metastasis, survival, and angiogenesis^25,46,47^. Akt and mTOR are both downstream targets of VEGFA, subsequently, we further examine whether ZFAS1 knockdown inhibited CRC tumor initiation and progression through inhibited EMT process and VEGFA/VEGFR2/Akt/mTOR signaling pathway, and our data showed ZFAS1 knockdown could significantly reduce the expression of VEGFA and changed EMT-related markers, reduced the activity of VEGFR2 and downstream Akt/mTOR signaling pathway, while antagomiR-150-5p and VEGFA both could restore the expression of VEGFA, the hallmarks of the EMT, the activity of VEGFR2 and downstream Akt/mTOR signaling pathway.

In summary, ZFAS1 expression was found to be increased in CRC cells and tissues, and was activated by transcription factor SP1. We also observed that ZFAS1, acting as a miR-150-5p sponge, promoted tumor growth, metastasis, and angiogenesis in CRC. Furthermore, we verified that ZFAS1 knockdown inhibited EMT process and inactivated VEGFA/VEGFR2/Akt/mTOR signaling pathway in CRC. Our data will provide new insights into the underlying mechanism of CRC progression and suggested that ZFAS1 might serve as a potential prognostic biomarker and a promising therapeutic target for CRC.

## Electronic supplementary material

Fig S1, S2, S3, S4, S5

Supplementary materials and methods

## Data Availability

All data generated or analyzed during this study are included either in this article or in the supplementary files.
