# Supplementary material for: SP1-induced lncRNA-ZFAS1 contributes to colorectal cancer progression via the miR-150-5p/VEGFA axis
Source: Cell Death Dis. 2018 Sep 24;9(10):982. doi: 10.1038/s41419-018-0962-6 (PMC6155123; doi:10.1038/s41419-018-0962-6)
Supplement: Supplementary file 1 — Fig S1, S2, S3, S4, S5 [file 41419_2018_962_MOESM1_ESM.docx]

**Supplementary Figure legends**

**Fig S1.** SP1 mRNA was upregulated in CRC. A, B. SP1mRNA was upregulated in CRC cells(A) and tissues(B). C. SP1 mRNA was positively correlated with ZFAS1 in CRC tissues. ****P*<0.001.
Fig S2. A-D. Representive amages of colony formation(A), wound healing(B, ×40),
transwell(C, ×200) and tube formation(D, ×100) in siZFAS1-1 group,
siZFAS1-2 group, scramble group.
Fig S3. A-D. representive amages of wound healing(A), transwell(B) and tube formation(C) in scramble group, siZFAS1-1 group, siZFAS1-1+antagomiR-NC group and siZFAS1-1+antagomiR-150-5p group.

Fig S4. The representive CT scans of lung metastatic foci of the nude mice(left), representative images of lungs from nude mice(middle) and the microscopic images of lung tissue sections stained by hematoxylin and eosin(right).

Fig S5. Representive amages of wound healing(A), transwell(B) and tube formation(C) after adding Ki8751 to HCT116 and HT29 cells.


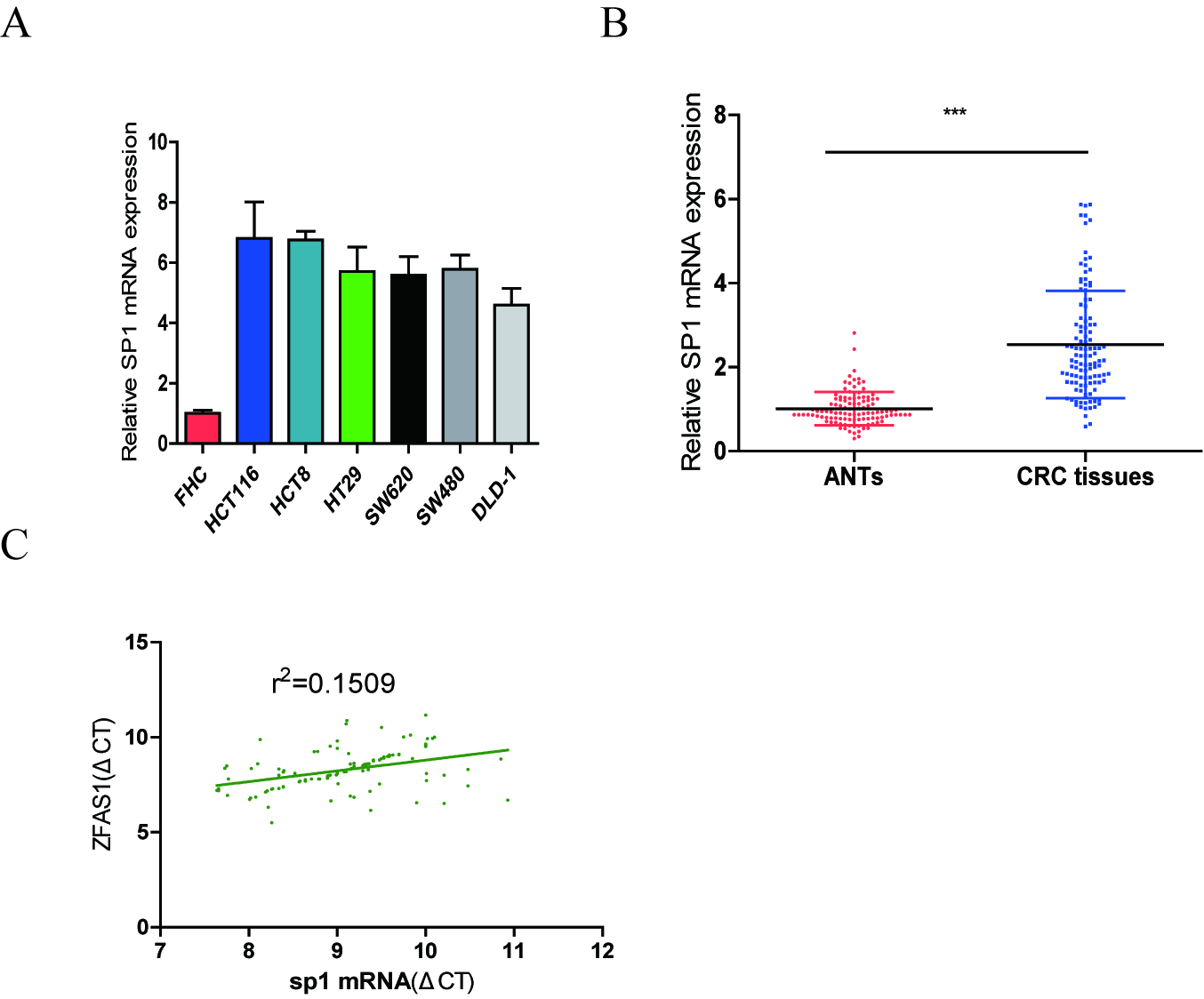


Fig S1


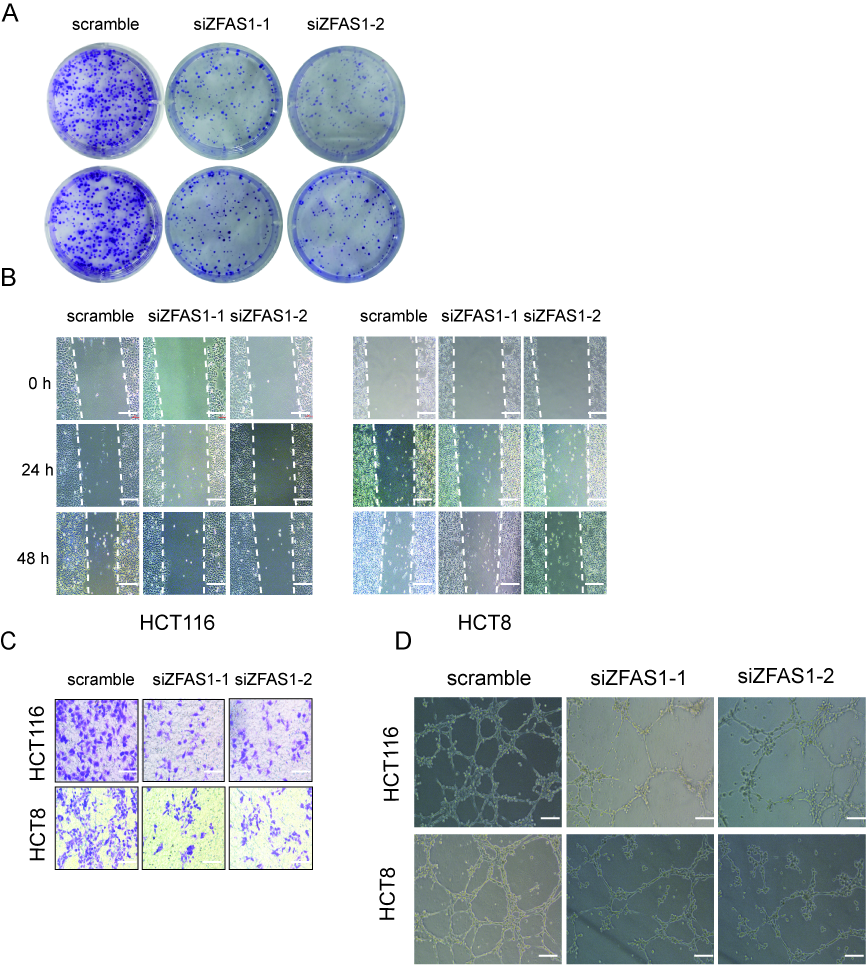


Fig S2


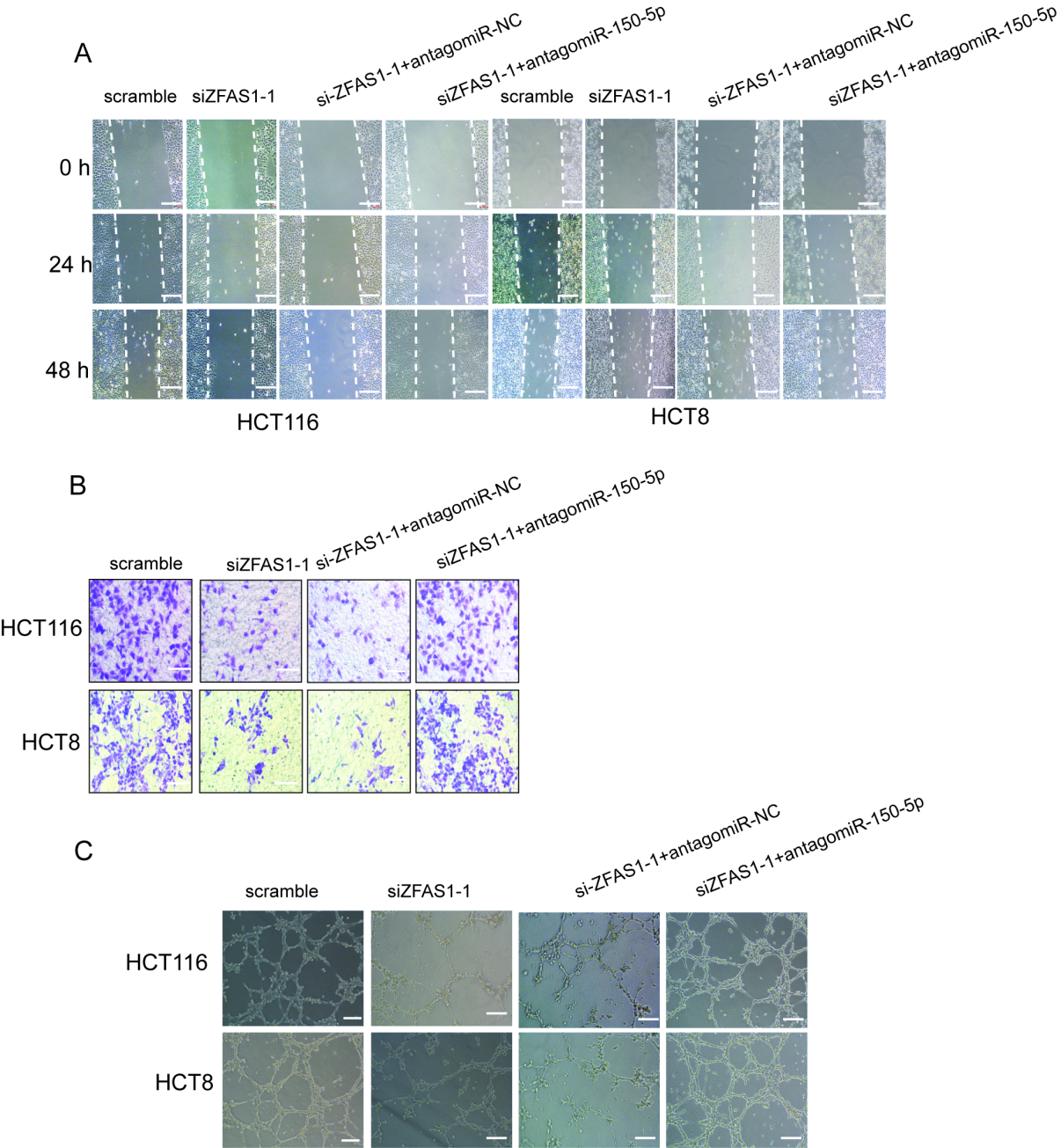


Fig S3


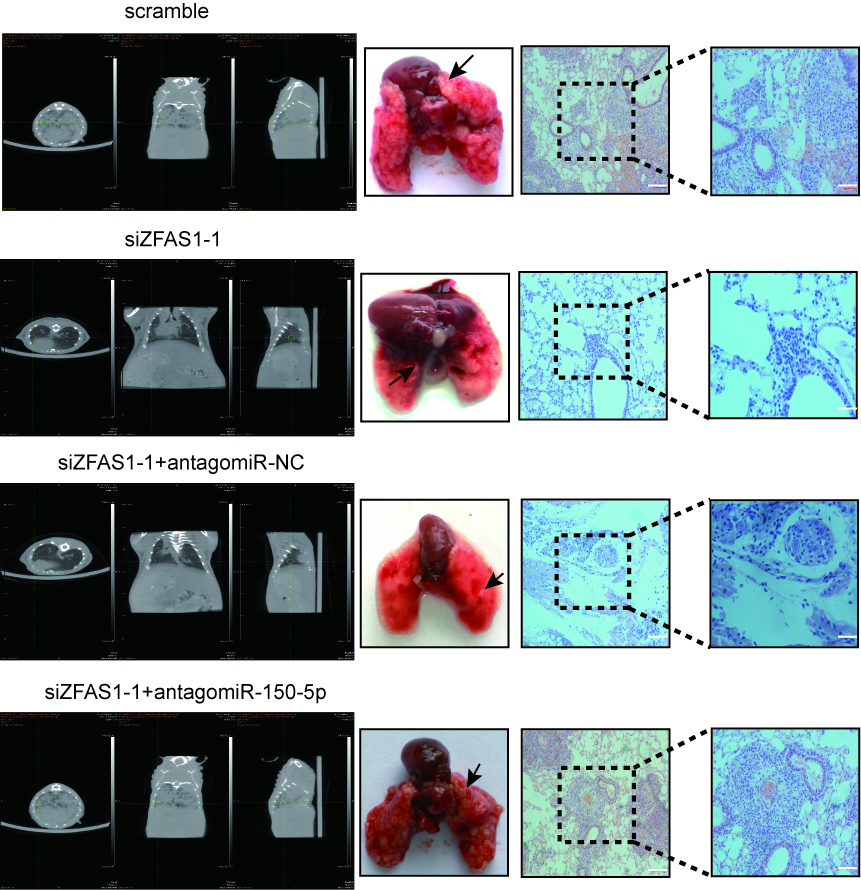


Fig S4


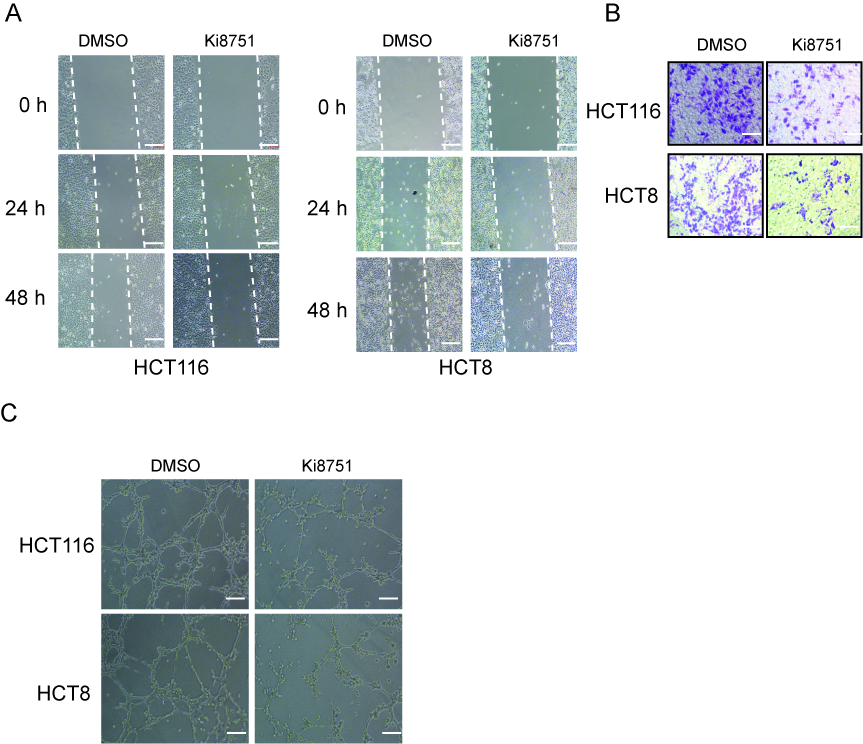


Fig S5
