# Supplementary material for: SP1-induced lncRNA-ZFAS1 contributes to colorectal cancer progression via the miR-150-5p/VEGFA axis
Source: Cell Death Dis. 2018 Sep 24;9(10):982. doi: 10.1038/s41419-018-0962-6 (PMC6155123; doi:10.1038/s41419-018-0962-6)
Supplement: Supplementary file 2 — Supplementary materials and methods [file 41419_2018_962_MOESM2_ESM.pdf]

Supplementary materials and methods

Cell proliferation assay

Cell Counting Kit-8(CCK-8, KeyGEN BioTECH, China) was performed to evaluate cell proliferation according to the manufacturer's instructions. Approximately 1×10^3 transfected cells were seeded into a 96-well plate. After 12h, 24h, 48h and 72h, 10μl CCK-8 test solution were added into a 96-well plate, and incubated for 3 h. The absorbance at 450nm was measured in a microplate reader(Infinite M200 Pro, Tecan).

Colony formation assay

CRC cell lines HCT116 and HCT8(2.5×10^3) were plated into a six-well plate, then cultured for 2 weeks after treatment. After fixing with 100% Methanol for 15 min and washing, fixed cells were then stained by 0.1% crystal violet staining solution (Beyotime, China).

Wound-healing assay

The wound-healing assays were performed to evaluate cell migration. Transfected and corresponding control cells were plated into a six-well plate(4×10^5 cells/ well), The cells were cultured with serum-free medium and incubated at 37℃ for 48 h, then the wound closure was detected by inverted microscope(Nikon, Japan) at ×40 magnification.

Transwell assay

The invasion of cells were evaluated by transwell assay, 3×10^5 cells were cultured in 200μl serum-free media in the upper chamber pre-coated with 100 μl 2% matrigel(BD Biosciences, USA). The lower chamber was filled with 500 medium containing 10% FBS. After incubation for 36 h, the non-invasion cells were wiped off in the upper chamber with cotton swabs, and the cells in the lower surface were fixed by methanol, then stained with 0.1% crystal violet(Beyotime, China). The invasion cells were counted under inverted microscope(Nikon, Japan) at ×200 magnification.

Tube formation assay

HVVECs cells(7×10^5) were suspended by the mixture of tumor-conditioned medium(TCM, 300μl) and DMEM containing 10% FBS(300μl), and then plated into a 24-well plate precoated with matrigel(200 μl per well, BD Biosciences, USA). tube

formation was observed after 6 h incubation at 37℃and was imaged with a computer-assisted inverted microscope(Nikon, Japan), the number of tube branches was counted by image J software.

Western blotting

The total proteins were extracted from cells with RIPA lysis buffer mixed with phenylmethyl sulfonylfluoride(PMSF), protein inhibitors and phosphatase inhibitors(KeyGEN BioTECH, China). Equal amount of proteins were separated with 10% SDS-PAGE gel and transferred to polyvinylidene difluoride(PVDF) membranes(Millipore, USA). The membranes were blocked with 5% bovine serum albumin(BSA) for 1.5 h and then incubated with primary antibodies: rabbit polyclonal anti-VEGFA(1:1000, ab46154, abcam , UK), anti-VEGFR2(1:1000, ab39256, abcam , UK), anti-phospho(Y1175)-VEGFR2(1:1000, ab194806, abcam , UK), ant-Akt(#9272, cell signaling technology, USA), anti-phospho(Ser473)-Akt(1:2000, #4060, cell signaling technology, USA), ant-mTOR(1:1000, ab2732, abcam , UK), anti-phospho(S2448)-mTOR(1:1000, ab84400 abcam , UK), rabbit anti-N-cadherin (1:1000, 22018-1-AP, proteintech, China), rabbit anti-E-cadherin (1:1000, 20874-1-AP, proteintech, China), rabbit anti-vimentin (1:1000,10366-1-AP, proteintech, China) and rabbit anti-GAPDH(1:10000, ab9485, abcam, UK). Proteins were then detected by enhanced chemiluminescence system(ECL) reagent(KeyGEN BioTECH, China) after incubation with secondary antibodies for 1 h at room temperature.

RNA isolation and quantitative RT-PCR

TRIzol Reagent (Invitrogen, USA) was performed to extract total RNA in accordance with manufacturer's instructions. MiR-150-5p expression was detected by a Hairpin-it^{TM} microRNA and U6 snRNA normalization RT-PCR quantitation kit(Genepharma, China). For ZFAS1 mRNA and VEGFA mRNA, complementary DNA(cDNA) was synthesized using PrimeScript^{TM} reagent kit with gDNA Eraser(Takara, Dalian, China) and qRT-PCR was analyzed using SYBR Premix Ex Taq kit(Takara, Dalian, China). The PCR primes for these genes were listed in supplementary table 2. The relative expression of miRNA or mRNA was analyzed

using $2^{-\Delta\Delta CT}$ method. All results were normalized to GAPDH or U6. The primers of ZFAS1, VEGFA mRNA and GAPDH are listed as follows.

ZFAS1	Forward: 5'-CCGGAGTGTGGTACTTCTCC-3'
	Reverse: 5'-CCAGAGGTCTCCAACGAAGA-3'
VEGFA	Forward: 5'-TGGCTCACTGGCTTGCTCTA-3'
	Reverse: 5'-ATCCAACTGCACCGTCACAG-3'
GAPDH	Forward: 5'-GGTGGTCTCCTCTGACTTCAA-3'
	Reverse: 5'-GTTGCTGTAGCCAAATTCGTTGT-3'

Tumor xenografts and tail vein injection experiments

All animal experiments were approved by the animal care Committee of Nanjing First Hospital, Nanjing Medial University(acceptance No. SYXK 20160006). 4-6 weeks BALB/c nude mouses were purchased from College of Veterinary Medicine Yang Zhou University. For xenografted tumor model, 1×10^7 HCT116 cells or HCT116 cells stably with ZFAS1 knockdown in 0.2 ml PBS were subcutaneously injected BABL/c nude mouses which were randomly divided into four group(eight mice per group). After tumor formation(day 6), 2 nmol antagomiR-150-5p or negative control (antagomiR-NC) were injected into tumors, the injection were performed 7 times at an interval of 2 days between each injection(day 6, 8, 10, 12, 14, 16, 18). The mice were sacrificed after the 2 days of ther last injection, and the volume of the tumors were calculated with the following equation: $V=0.5\times(\text{length} \times \text{width}^2)$.

For metastasis experiments, 2×10^6 cells in 0.2 ml PBS were injected into the tail vein of nude mouses which were randomly divided into four groups(eight mice per group). After 8 weeks of injection. Computed Tomography(CT) scan were performed to detect the lung metastasis of mouses. After CT scan, mice were sacrificed, and their lungs were removed and stained by Hematoxylin and Eosin (HE) Staining.

Chicken chorioallantoic membrane(CAM) assay

All animal experiments were approved by the animal care Committee of Nanjing First Hospital, Nanjing Medial University(acceptance No. SYXK 20160006). The specific pathogen-free fertilized chicken eggs were incubated in an 55% -65% humidified atmosphere at 37℃ for 6 days. Then a window of approximately 1.0 cm^2 was made

on each egg into the shell, and conditioned medium(CM) from CRC cells in the siZFAS1-1 group, scramble group, siZFAS1-1+antagomiR-NC group, siZFAS1-1+antagomiR-150-5p group was mixed with matrigel, and then deposited in the center of each egg. The CAM results were collected with photographic document after 4 days . At least 8 viable embros were examed for each treatment , and the angiogenesis effect of each egg was quantified by counting the number of blood vessel branches.

The sequence of siZFAS1, siSP1, agomiR-150-5p, agomiR-NC, antagomiR-150-5p and antagomiR-NC

scramble	UUCUCCGAACGUGUCACGUdTdT
siZFAS1-1	AGCGGTTTGGTGCGGTGTGAAGCGCGACAT
siZFAS1-2	AGCCATCTTTGGTTATATAAGGGAGGTTC
siZFAS1-3	CCACGTGCAGACATCTACAACCTTCGATC
siZFAS1-4	AGTCTGCCTTGTAACAGAAACTGGCGATG
siSP1-1	CAGCGUUUCUGCAGCUACCUUGACU
SiSP1-2	GACAGGUCAGUUGGCAGACUCUACA
agomiR-150-5p	5’-UCUCCCAACCCUUGUACCAGUG-3’
	5’-CUGGUACAAGGGUUGGGAGAUU-3’
agomiR-NC	5’-UUCUCCGAACGUGUCACGUTT-3’
	5’-ACGUGACACGUUCGGAGAATT-3’
antagomiR-150-5p	CACUGGUACAAGGGUUGGGAGA
antagomiR-NC	CAGUACUUUUGUGUAGUACAA

The primes of qChIP

E1 region	Forward: 5’-GGAAACTGTGACTACATTTGGCA-3’
	Reverse: 5’-CAAACTCTAAATACTGAGCCTGCG-3’
E2 region	Forward: 5’- GAGCGCCAAGGCTGTGAGGT-3’
	Reverse: 5’-CTCTTCTACAGAGCACCTCGGC-3’
E3/E4 region	Forward: 5’-GCACTTTCGGTTTCCGTTCCC3’
	Reverse: 5’-CCTCGTGCTCTCCACCCTGG-3’
